# Evaluation of intestinal immune responses to *Entamoeba dispar* using the first domestic clinical isolate in Japan

**DOI:** 10.1371/journal.pntd.0014507

**Published:** 2026-08-06

**Authors:** Yumiko Saito-Nakano, Akira Kawashima, Seiki Kobayashi, Koji Watanabe, Tetsuro Kawano-Sugaya, Shinji Izumiyama, Kumiko Nakada-Tsukui

**Affiliations:** 1 Department of Parasitology, National Institute of Infectious Diseases, Japan Institute for Health Security, Tokyo, Japan; 2 AIDS Clinical Center, National Center for Global Health and Medicine, Japan Institute for Health Security, Tokyo, Japan; 3 Department of Parasitology, Tokai University School of Medicine, Isehara, Japan; 4 Department of Biomedical Chemistry, Graduate School of Medicine, The University of Tokyo, Tokyo, Japan; Advanced Centre for Chronic and Rare Diseases, INDIA

## Abstract

**Background:**

*Entamoeba dispar* is a non-pathogenic sibling species of *Entamoeba histolytica*, yet the mechanisms that enable *E. dispar* to persist in the intestine without causing disease remain poorly understood. Experimental models for dissecting intestinal immune responses to *E. dispar* have been limited. In Japan and other East Asian countries, *E. histolytica* infection is relatively common among men who have sex with men, whereas *E. dispar* infection has rarely been identified.

**Methodology/Principal findings:**

We report the first domestically acquired and culture-established *E. dispar* infection in Japan. A clinical strain, NA442, was isolated from fecal cysts of an HIV-positive patient without gastrointestinal symptoms and was maintained in stable monoxenic culture with *Pseudomonas putida*. Phylogenetic and tRNA-linked STR analyses confirmed the species identity and revealed genetic similarity to a Thailand-derived genotype.

Using NA442, we established a murine cecal infection model to characterize host immune responses. Infection induced detectable secretion of lipocalin-2 in stool, indicating epithelial stress; however, CXCL-1 and myeloperoxidase levels in cecal tissue remained at baseline, and no neutrophil infiltration or tissue destruction was observed. NA442 colonized the murine cecum for up to three weeks but elicited only mild epithelial thickening without the hallmark inflammatory signatures associated with *E. histolytica*.

**Conclusions/Significance:**

This study provides the first in vivo evidence that *E. dispar* colonization triggers limited mucosal inflammation despite successful short-term persistence in the intestine. The identification of NA442 as the first domestic *E. dispar* case in Japan suggests the parasite may circulate unnoticed within the population. The NA442 strain establishes a valuable experimental tool for comparative studies of pathogenic versus non-pathogenic *Entamoeba* species and contributes to understanding the determinants of intestinal virulence and host immune modulation.

## Author summary

*Entamoeba dispar* is a close relative of the pathogenic parasite *Entamoeba histolytica*, yet *E. dispar* is regarded as non-pathogenic and generally does not cause intestinal disease. Although the two species are genetically similar, the host immune responses they elicit differ markedly, and the mechanisms that allow *E. dispar* to persist without symptoms remain poorly understood. In Japan and other East Asian countries, *E. histolytica* infection is relatively common among men who have sex with men, whereas *E. dispar* infection is rare. This study reports the first *domestically acquired* case of *E. dispar* infection in Japan, highlighting the possibility that this parasite may circulate unnoticed in the local population. We established the clinical strain NA442 in culture and employed a murine cecal infection model to characterize the host immune response. *E. dispar* triggered an epithelial stress response, with limited neutrophil-associated responses and no evidence of tissue damage. These findings advance our understanding of how non-pathogenic *Entamoeba* interacts with the host intestine and highlight the biological distinction between *E. dispar* and *E. histolytica*. Our work also raises awareness of the potential presence of *E. dispar* within Japan and provides a valuable platform for future studies of host–parasite interactions and amoebiasis epidemiology.

## Introduction

Amebiasis caused by the fecal–orally transmitted protozoan parasite *Entamoeba histolytica* has been recognized as a sexually transmitted infection in high-income countries [1,2]. In these settings, HIV-positive men who have sex with men (MSM) represent a major risk group. Interestingly, however, the epidemiological pattern differs in East Asian countries, including Japan, where MSM are predominantly infected with *E. histolytica*, whereas this population is more commonly colonized by the non-pathogenic sibling species *Entamoeba dispar* in Western countries [3,4].

Host genetic studies have linked leptin receptor Q223R mutation to susceptibility to intestinal amebiasis, highlighting the importance of neutrophil recruitment in restricting *E. histolytica* colonization in the intestine, potentially due to impaired leptin-dependent neutrophil chemotaxis [5,6]. Although *E. histolytica* is a human parasite, murine cecal infection model was established in 2002 following the development of several experimental intestinal models including closed ileal loop models, colonic loop models, proximal common ligation models, and surgically isolated intestinal segment models [7]. This intracecal inoculation model has since been widely used to dissect mucosal immune responses, demonstrating that parasite–epithelial interactions induce inflammatory cytokines and chemokines, including CXCL1/2, which drive neutrophil recruitment [8,9]. However, comparable intestinal infection models for *E. dispar* have not been established.

Comparative studies of *E. histolytica* and *E. dispar* have highlighted the importance of hydrolytic enzymes (such as cysteine proteases) and adhesion molecules (such as the Gal/GalNAc-specific lectin) in virulence. In particular, the lack of EhCP-A5 expression and the lower surface abundance of the Gal/GalNAc-lectin intermediate subunit (Igl) in *E. dispar* are associated with its markedly reduced pathogenicity [10,11]. Despite several attempts, stable intestinal colonization for *E. dispar* has remained difficult to achieve in murine models [12]. Unlike *E. histolytica*, most *E. dispar* strains require symbiotic intestinal bacteria for proliferation [13], and the xenic SAW strains are commonly used [14]. Therefore, freshly established axenic or monoxenic *E. dispar* strains are needed to enable detailed analyses of intestinal colonization and host immune responses.

In this study, we report the establishment of the first domestic *E. dispar* strain in Japan. This strain, designated NA442, was isolated from the stool of an HIV-positive individual and successfully maintained as a monoxenic culture. Its identity as *E. dispar* was confirmed by PCR and sequence analysis. Using this fresh isolate, we further investigated host–parasite interactions in a murine cecal infection model in a substrain of C57BL/6 mice. To our knowledge, this is the first report to characterize a Japanese *E. dispar* strain and to examine *E. dispar*–induced immune responses in a murine cecum model. The establishment of strain NA442 provides an important experimental resource for understanding immune responses to *E. dispar* and offers a comparative framework for elucidating the determinants of pathogenicity in *E. histolytica*.

## Methods

### Ethics statement

The study was conducted in accordance with the Declaration of Helsinki and approved by the Ethics Committee of National Institute of Global Health and Medicine (NCGM-S-004658–02). Written informed consent was obtained from the participant. All animal experiments followed the institutional regulations of the National Institute of Infectious Diseases (NIID), and were approved by the Institutional Animal Care and Use Committee of NIID (Approval No. 123041). Experiments complied with national guidelines for animal welfare, including the 3R principles, and appropriate measures were taken to minimize pain, distress, and to ensure humane euthanasia.

### Cell culture and reagents

*E. dispar* NA442 strain was cultured in YIMDHA-S medium or BI-YIMDHA medium supplemented with 15% of adult bovine serum, *Crithidia fasciculata*, *Pseudomonas aeruginosa*, or *Pseudomonas putida* in the presence of antibiotics (penicillin, streptomycin, and amphotericin B, Wako) [15]. *E. histolytica* strain HM-1:IMSS cl6 passaged through hamster liver, called ALA strain [16], was maintained in BI-YIMDHA medium supplemented with 15% of adult bovine serum and *C. fasciculata* in the presence of antibiotics.

### Stool examination and diagnosis

Fresh stool samples were processed within two hours. Concentrated stool smears were examined microscopically, and amebiasis was diagnosed using an *E. histolytica* antigen test (*E. histolytica* QUIK CHEK, TechLab) and an *E. histolytica*–specific quantitative PCR assay [17].

To identify *Entamoeba* species, DNA was extracted from 200 µL of the culture suspension using a QIAamp DNA Stool Mini kit (QIAGEN). PCR targeting the 1.8-kbp rRNA gene was performed using *Entamoeba* genus-specific universal primers: Enta_univF_niced (5′-CTGCCAGTATTATATGCTGATGTT-3′) and Enta_univR_niced (5′-TCTCCTTCCTCTAAATAAGGAGATTTA-3′) [18]. PCR was performed using PrimeSTAR GXL DNA polymerase (Takara) with the following cycling conditions: 98 °C for 10 sec; 40 cycles of 98°C for 10 sec, 60°C for 15 sec, and 68 °C for 15 sec.

### Establishment of *E. dispar* clinical strain

Amoeba cysts were partially purified from approximately 200 mg of stool of the patient. The sample was washed twice with Milli-Q water by centrifugation at 450 × g for 3 minutes to remove debris and commensal bacteria. The pellet was treated with 0.05 N HCl for 8 minutes at room temperature to kill concomitants according to a previously reported protocol [19]. After further washing, the cyst-rich fraction was inoculated into YIMDHA-S medium [15,20] containing Antibiotic-Antimycotic (Gibco, cat. no. 15240062) in the presence of *C. fasciculata* (ReF-1: PRR, ATCC50083), for inducing excystation and culturing the trophozoites. After establishing monoxenic culture in YIMDHA-S medium supplemented with *C. fasciculata*, *P. aeruginosa* (strain PA: KEIO) was adopted as the growth associate for monoxenic cultivation of the *E. dispar* isolate to achieve more stable growth and higher yield [21].

For animal experiments, BI-YIMDHA-S medium was used instead of YIMDHA-S; in BI-YIMDHA-S, the 3% yeast extract of YIMDHA-S is replaced with an equal concentration of Biosate Peptone (Gibco, cat. no. 211862). Avirulent *P. putida* (NBRC 109348) was chosen as a growth associate instead of *P. aeruginosa*, and 2 mM sodium pyruvate (a growth-promoting substance for *E. dispar*) (Sigma Aldrich, cat. no. S8636) was added together with *P. putida*.

### Phylogenetic analysis

The 18S rRNA sequences of *Entamoeba* species were retrieved from GenBank. They were aligned by the MAFFT-linsi method in MAFFT v7.526 [22]. We selected well-aligned regions using trimAl v1.5.rev0 [23] with -automated 1 option. Phylogenetic analyses were performed by IQ-TREE version 3.0.1 [24] with the following options (-m MFP -B 1000 -alrt 1000 -bnni -T AUTO). The result was visualized using FigTree v1.4.4 (https://github.com/rambaut/figtree). The 18S rRNA sequence of NA442 was deposited in GenBank database under accession number LC901119.

### Genotyping

Primer sequences for *E. histolytica* and *E. dispar* common transfer RNA (tRNA) short tandem repeat (STR) region Asp-Ala (D-A) were identified from whole-genome information of the NA442 strain. The primer sequences are as follows; D-A5 (EhR1) 5’-CTGGTTAGTATCTTCGCCTGT-3’, D-A3 (EhR2) 5’-GCTACACCCCCATTAACAAT-3’ [25]. The genome information of NA442 strain is available in GenBank (accession GCA_057946545.1). This primer set amplified a 1,250-bp genomic region, of which 459-bp corresponded to STR D-A locus [25]. We used the sequence for STR analysis.

### Experimental intestinal amebiasis model in murine cecum

Six-week-old male C57BL/6NCrSlc mice (Japan SLC Inc.) were maintained under conventional conditions at JIHS-NIID. All procedures were approved by Institutional Animal Care and Use Committee. For intracecal inoculation, mice were anesthetized with isoflurane, the abdomens were shaved and incised, and the cecum was exteriorized as previously reported [7]. Trophozoites (1 × 10^6^ or 1 × 10^5^ in 200 μL BI-YIMDHA medium) were injected into the cecum apex. Sham controls received 100 μL of BI-YIMDHA medium. The cecum was blotted, and the peritoneum and the skin were sutured.

### PCR amplification to detect *Entamoeba* from murine stool

Stool samples were collected daily from mice for up to 7 days post-surgery. DNA was extracted from 3–5 stool pellets using a QIAcube and the QIAamp Fast DNA stool Mini Kit (Qiagen). Infection was assessed by PCR targeting the 18S rRNA gene (GenBank: AB282658) using a primer set common to *E. histolytica* and *E. dispar* (EntaF3 5’- ATCCATGATCGCTATAAGATGCACGAGAG-3’; EhR-4 5’-CCATAAACTCAAGATTTCTCTTTAAGTTCTGAACAA-3’).

### Pathology and evaluation of lesion area

Murine cecum samples were preserved in 10% formalin solution. The preparation of paraffin sections and periodic acid-Schiff (PAS) staining for the tissue samples was conducted by Biopathology Institute Co., Ltd. Anatomically comparable regions of the cecum were selected for quantitative analysis to minimize variability due to sectioning position. The area of red-stained goblet cells, length of mucosa, and deep-stained trophozoites were measured using a microscope IX73 equipped with Visualix Pro2 Metrics imaging system (Evident). Mucosal thickness was measured at 30 independent sites per mouse across PAS-stained cecal sections, and data from three mice (total 90 measurement points) were included in the analysis. Trophozoites, which stain a deep purple, are recognizable in PAS staining [2]. To visualize infiltrated neutrophils, paraffin sections were stained with an anti-Ly6G antibody (Abcam, ab238132; 1:100 dilution), followed by horseradish peroxidase-diaminobenzidine (HRP-DAB) detection and hematoxylin counterstaining. Immunohistochemical staining was performed by Biopathology Institute Co., Ltd.

### Cytokine detection by ELISA

Stool samples were resuspended in 1 mL PBS containing 0.1% Tween 20 and centrifuged 23,000 × *g* for 10 min at 4°C [9]. The clear supernatant was used for Lipocalin-2 ELISA (DY1857, R&D systems). Resected cecal tissues were bead-beaten for 1 min in 250 μl of lysis buffer I [1 × cOmplete mini protease inhibitor cocktail (Roche), 5 mM HEPES] followed by addition of 250 μl of Lysis buffer II (2% Triton X-100 in lysis buffer I) and inverted gently. After incubation on ice for 30 min, samples were centrifuged at 13,000 × *g* for 5 min at 4°C, and the supernatant were collected. Protein concentration was assessed by a *DC* protein assay kit (Bio-Rad). CXCL1, MPO and IL-10 were quantified using ELISA kits ab216951 (Abcam), EK0493 (BosterBio), KE10103 (Proteintech), respectively. All assays were performed according to the manufacturers’ instructions, and values were normalized to total protein content.

### Statistical analysis

ANOVA was used for differences among multiple groups. Student’s t-test, log-rank test, and Fisher’s LSD test were used as appropriate for comparing variables in two groups. A P-value < 0.05 was considered statistically significant.

## Results

### Clinical case presentation

The patient was a man in his 40s with HIV-1 infection for 15 years, admitted with a third episode of *Pneumocystis* pneumonia (PCP) after four years of treatment interruption. He had previously maintained viral suppression on cART, with a CD4 count of 150 cells/µL at his last routine visit (normal reference range: 500–1,500 cells/µL; below the critical threshold of 200 cells/µL for opportunistic infections). His medical history included treated syphilis and chronic hepatitis B virus carriage. He identified as bisexual, reported anal intercourse and last sexual activity approximately five years ago with a female partner. He worked as a hospice manager, lived alone in Tokyo, and had regular contact with two domestic dogs owned by his partner. He had no recent overseas travel history for more than 10 years, and no history of commercial sex work or injection drug use.

On admission, he presented with fever, hypoxia, and oral candidiasis. Chest CT showed bilateral ground-glass opacities, whereas abdominal CT revealed no abnormalities, and he reported no gastrointestinal symptoms. PCR of induced sputum confirmed *Pneumocystis jirovecii* (1,000 copies/mL). Laboratory tests demonstrated a CD4 count of 68 cells/µL and an HIV-RNA level of 428 copies/mL, indicating advanced immunosuppression.

During routine screening for opportunistic infections, stool ova and parasite examination performed on Day 4 incidentally detected *Entamoeba* cysts.

### Stool examination and diagnosis of *E. dispar*

**Fig 1:**
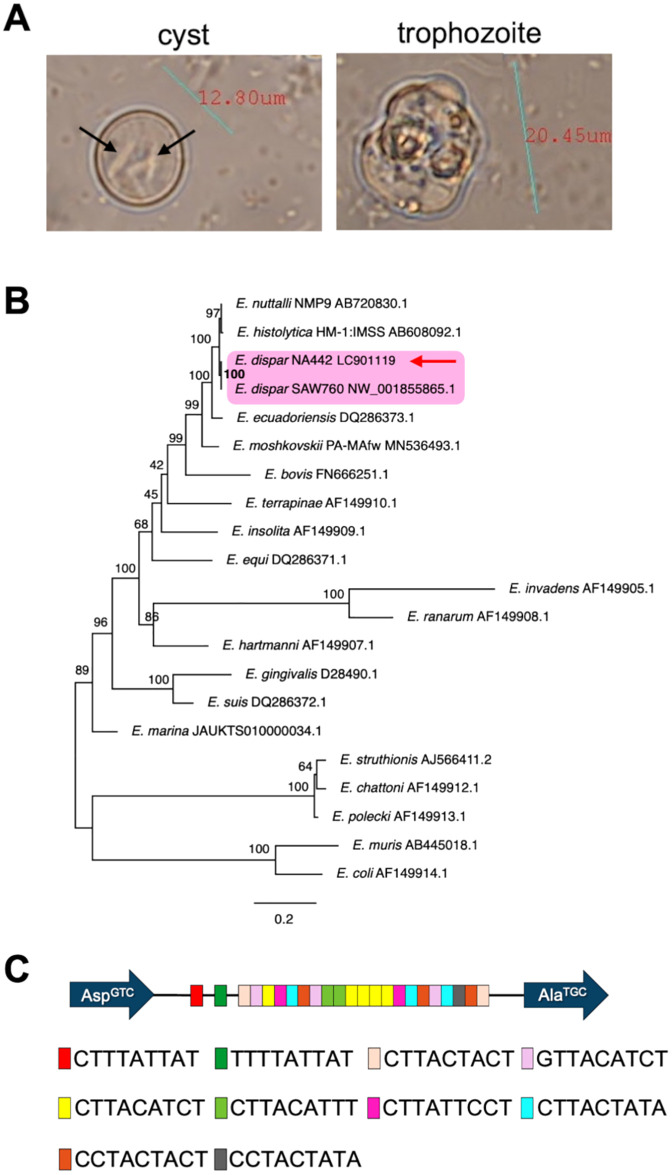
Genetic background of Japanese isolate of *E. dispar* NA442 strain. (A) Representative images of a cyst (left) and a trophozoite (right) of *E. dispar* NA442 strain. Arrows indicate chromatoid bodies. (B) Phylogenetic tree based on rRNA sequences from *E. dispar* NA442 and twenty other species retrieved from GenBank. (C) Schematic representation of polymorphism in D-A locus of tRNA-linked short tandem repeat region.

Direct smear microscopy revealed cysts morphologically consistent with the *Entamoeba histolytica/dispar/moshkovskii* complex. To diagnose the amebiasis, an *E. histolytica* antigen test (QUIK CHEK) and an *E. histolytica*–specific qPCR [17] were performed and were both negative. Subsequent microscopic re-evaluation identified cysts with uniform morphology and clearly defined trophozoite plasma membranes. The cysts measured 13.1 ± 0.94 µm in diameter and contained four chromatoid bodies, while trophozoites measured 19.8 ± 1.24 µm. These sizes were smaller than those reported for *E. histolytica* [26] and active proliferation of trophozoites immediately after isolation, collectively supporting identification as *E. dispar* (Fig 1A).

Because the patient’s risk for *E. dispar* infection was apparently low, we aimed to identify *Entamoeba* species. A 1.8-kb rRNA gene fragment was amplified using genus-specific primers and subjected to Sanger sequencing. BLASTn analysis of the forward sequence showed 99.78% identity with established *E. dispar* rRNA sequences (e.g., AB282661.1, KP722584.1) with 100% query coverage, and the reverse sequence exhibited up to 99.89% identity with *E. dispar* strains (e.g., AB282661.1, LC014025.1). In contrast, both sequences showed <97% identity and multiple mismatches when compared with *E. histolytica* or *E. moshkovskii*, confirming species-level identification as *E. dispar*. Phylogenetic analysis further demonstrated that the patient-derived rRNA gene sequence formed a monophyletic cluster with *E. dispar* SAW760 (Fig 1B).

The patient had no history of international travel in ten years, indicating that this represents the first documented domestic case of *E. dispar* infection in Japan. We designated the isolate as strain NA442. To characterize NA442 further, we analyzed the genotype of the tRNA-linked STR at the D-A locus [25]. The STR profile matched that of Ed10DA, a genotype detected in only one of 47 *E. dispar*–positive stool samples from 1,233 Thai schoolchildren in a previous study (Fig 1C) [25]. This suggests a correlation between the strain and those in Thailand.

### *E. dispar* was retained in murine cecum for up to three weeks

**Fig 2:**
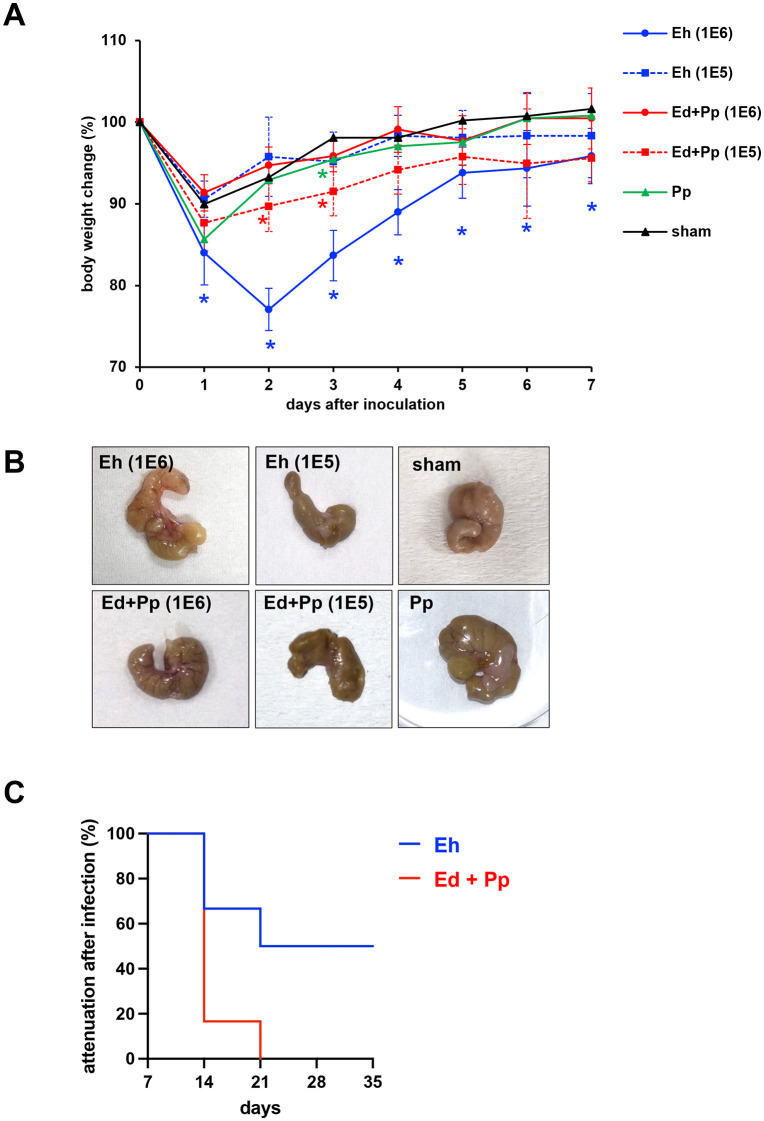
*E. dispar* NA442 colonizes the mouse cecum but is eliminated more rapidly than *E. histolytica.* (A) Changes in body weight were monitored in five successfully infected mice per group. C57BL/6NCrSlc mice were intracecally inoculated with 1 × 10^6^ cells of *E. histolytica* (blue line), 1 × 10^5^ *E. histolytica* (dotted blue line), 1 × 10^6^ cells of *E. dispar* (red line), 1 × 10^5^ *E. dispar* containing *P. putida* (dotted red line), *P. putida* (green line), or medium alone as a sham control (black line). Statistical comparisons were performed between each infected group and the sham-operated control at the corresponding time points using Student’s *t*-test. *p < 0.05. (B) Gross inspection of the cecum on day 7 post-challenge. (C) *E. dispar* NA442 was eliminated within 3 weeks. C57BL/6NCrSlc mice were inoculated with 1 × 10^6^ cells of *E. histolytica* (blue line) or 1 × 10^5^ *E. dispar* containing *P. putida* (red line). The time course of infection was monitored by PCR detection of the parasites in stool samples, starting from day 7 post-infection (P = 0.039, log-rank test).

Next, we utilized this newly established *E. dispar* clinical isolate to examine its infectivity in the murine cecal model. Infection outcomes were compared among mice challenged with *E. histolytica or dispar* NA442 co-cultured with *P. putida* at 1 × 10^5^ (1E5) and 1 × 10^6^ (1E6) cells per mouse, and control groups (Fig 2A). Body weight monitoring for seven days revealed significant weight loss in 1 × 10^6^ but not in 1 × 10^5^ *E. histolytica*–infected mice (Fig 2A, blue solid and dotted lines). Furthermore, mice inoculated with 1 × 10^6^ and 1 × 10^5^ *E. dispar* (red solid and dotted lines), *P. putida* alone (green line), or sham controls (black line) showed comparable trajectories, although 1 × 10^5^ *E. dispar*–challenged mice displayed slightly delayed recovery with significant differences on days 3–4. Gross examination of the cecum on day 7 showed preserved tissue morphology in *E. dispar*– and 1 × 10^5^ *E. histolytica*–inoculated mice, whereas 1 × 10^6^ *E. histolytica* infection induced epithelial swelling and luminal obstruction (Fig 2B).

PCR detection from fecal samples indicated that 70% (7/10) of mice inoculated with 1 × 10⁶ *E. histolytica* trophozoites remained positive at day 7, while all mice given a 10-fold lower dose (1 × 10^5^) were negative, demonstrating a threshold of approximately 1 × 10⁶ cells for stable colonization (Table 1). Notably, inoculation with 1 × 10^5^ and 1 × 10^6^ *E. dispar* trophozoites resulted in a similar positivity rate (60%) to the high-dose *E. histolytica* group, indicating efficient initial retention of *E. dispar*.

**Table 1:** Murine susceptibility to *E. histolytica* and *E. dispar* NA442.

Mouse strain	*Entamoeba* strain	inoculated cell number	infection rate (%)*
C57BL/6NCrSlc	*E. histolytica*	1 × 10^6^	7/10 (70%)
		1 × 10^5^	0/10 (0%)
	*E. dispar* NA442	1 × 10^6^	3/5 (60%)
		1 × 10^5^	6/10 (60%)

*, Infection rate was determined by PCR targeting *Entamoeba* genus-specific rRNA region using DNA purified from feces on day 7 post-infection.

Long-term monitoring up to 35 days demonstrated that 70% of *E. histolytica*–infected mice remained positive at day 14 and 50% remained positive at day 35 (Fig 2C). In contrast, 1 × 10^5^ *E. dispar* detection declined steadily: 20% were positive at day 14, and all mice became negative by day 21. Thus, NA442 persisted in the murine cecum for up to three weeks.

These results indicate that *E. dispar* NA442 shows stable short-term colonization without pathogenic features, supporting its utility as a model strain for investigating commensal behavior of *E. dispar*.

### *E. dispar* infection caused tissue thickening but no evidence for inflammation

**Fig 3:**
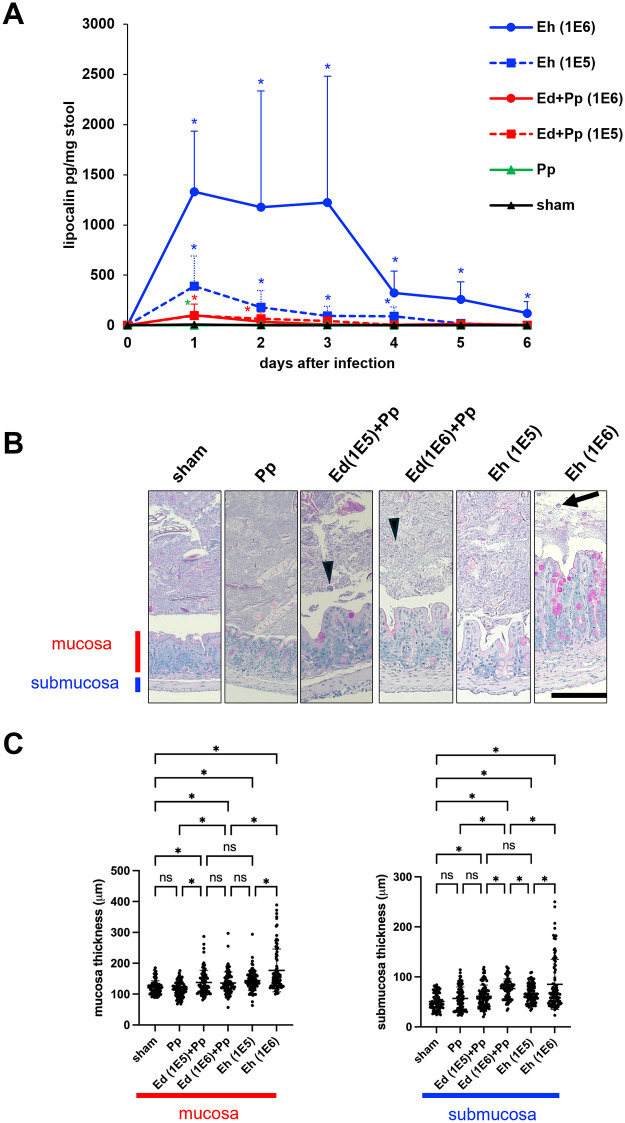
The early inflammatory response was triggered by *E. dispar.* (A) The fecal level of lipocalin-2 was measured for the assessment of neutrophil-mediated cecum inflammation (n = 5 per group). Statistical comparisons were performed between each infected group and the sham-operated control at the corresponding time points using Student’s *t*-test. *p < 0.05. (B) The representative PAS-stained section on day 7. Trophozoites of *E. dispar* (arrowheads) in the lumen and *E. histolytica* on the ulcerated epithelial surface (an arrow) were shown. Scale bar, 500 µm. Red and blue lines show mucosal and submucosal layers, respectively. (C) Mucosal and submucosal thicknesses were calculated from a total of 90 sections obtained from three independent experiments. Statistical comparisons between the indicated groups were performed using one-way ANOVA followed by the uncorrected Fisher’s LSD test. *p < 0.05.

To understand the molecular basis of immune response caused by *E. dispar* infection, we examined lipocalin-2 secretion, histology, and chemokine production [9]. Fecal samples from mouse with indicated treatment were collected for 7 days post-surgery and the amount of lipocalin-2 was quantified using an ELISA (Fig 3A). Of note that, in this experiment, we examined two different cell counts for *E. histolytica* and *E. dispar* to clarify the dose dependency. Amount of lipocalin-2 in stool was significantly high in 1 × 10^6^ *E. histolytica*–infected mouse up to 4 days post-surgery. In *E. dispar* infections, there was a lesser amount of lipocalin-2 secretion, however, significantly higher compared to sham control on days 1 and 2 post-surgery. These results demonstrate that the same count of *E. histolytica* and *E. dispar* cells induced an epithelial stress response, and infection of *E. histolytica* induces higher level of lipocalin than that of *E. dispar*.

Cecal tissues at day 7 post-infection were subjected to histopathology, and PAS staining visualized trophozoites as purple and host goblet cells as red (Fig 3B). Trophozoites of *E. histolytica* (an arrow) and *E. dispar* (arrowheads) were observed within the intestinal contents. Significant increases in mucosal and submucosal thickness were observed in 1 × 10^6^ *E. histolytica*–challenged mice, and thickening was also observed in *E. dispar*–challenged mice, which was statistically different from sham-infected and *P. putida* alone controls (Fig 3C).

**Fig 4:**
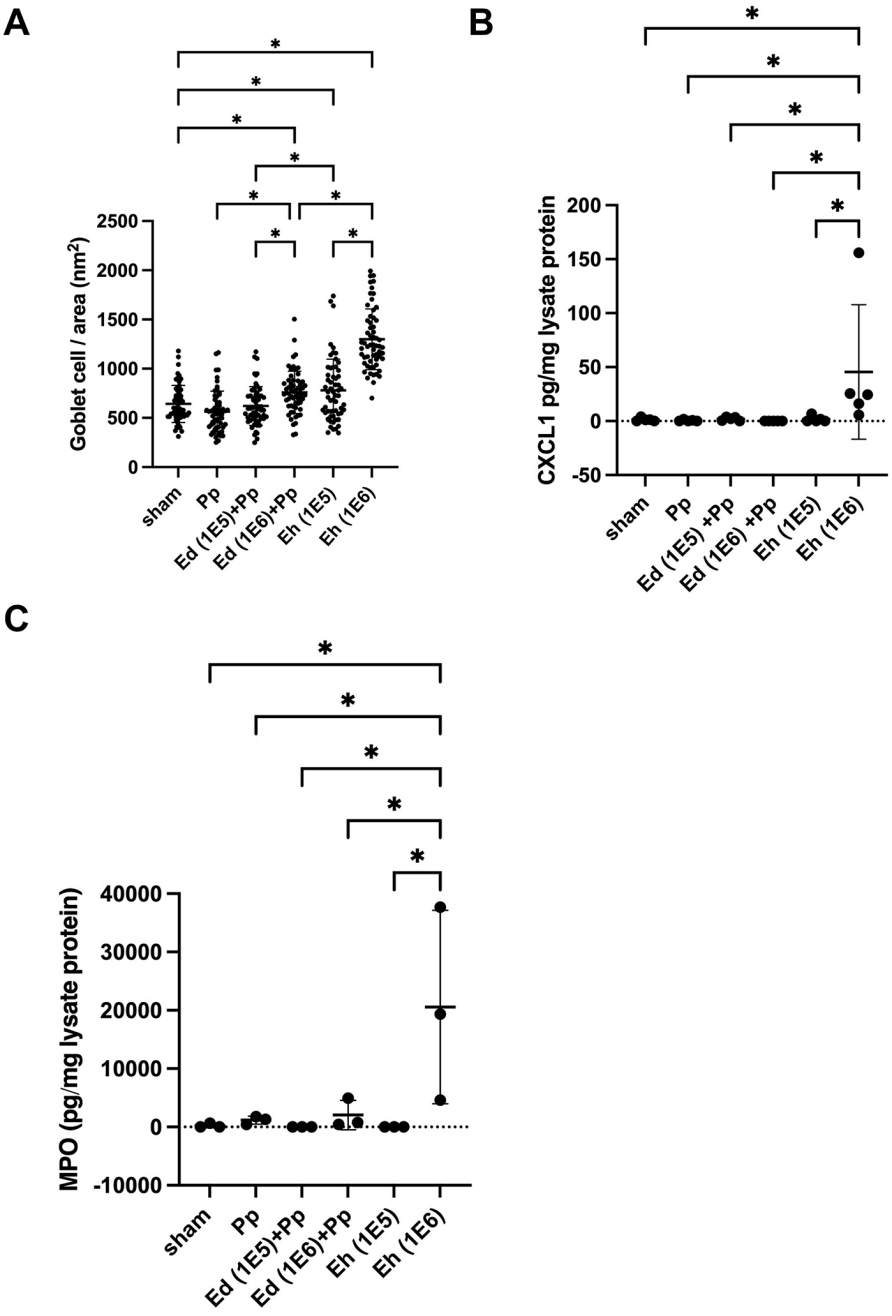
*E. dispar* did not evoke mucin and CXCL1 cytokine production. (A) The number of red-stained goblet cells was counted and normalized to the area of PAS-stained sections shown in Fig 3B. Mean values of a total of 90 sections obtained from three independent experiments are shown. Statistical comparisons between the indicated groups were performed using one-way ANOVA followed by the uncorrected Fisher’s LSD test. *p < 0.05. (B) CXCL1 levels were measured in cecal tissue lysates by ELISA and normalized to total protein concentration (n = 5 mice per group). Statistical comparisons between each group and the 1 × 10^6^ cells of *E. histolytica*–infected group were performed using one-way ANOVA followed by the uncorrected Fisher’s LSD test. *p < 0.05. (C) MPO levels were measured in cecal tissue lysates by ELISA and normalized to total protein concentration (n = 3 mice per group). Statistical comparisons between each group and the 1 × 10^6^ cells of *E. histolytica*–infected group were performed using one-way ANOVA followed by the uncorrected Fisher’s LSD test. *p < 0.05.

In contrast, the number of red-stained goblet cells at day 7 inoculation (Fig 3B) was increased only in the *E. histolytica*–infected cecum. Statistical significance between *E. dispar*–infected cecum and control groups was not observed (Fig 4A).

C-X-C motif (CXC) chemokine ligand 1 (CXCL1), a mouse homologue of human IL-8, is a key chemokine involved in neutrophil activation and inflammation in amebic colitis [27]. Mice infected with 1 × 10^6^ *E. histolytica* showed elevated level of CXCL1 as previously reported (Fig 4B) [27], while no induction of CXCL1 was observed in mice infected with 1 × 10^5^ *E. histolytica* and *E. dispar* (Fig 4B). Neutrophil myeloperoxidase (MPO) in cecum, an indicator of neutrophil infiltration was determined (Fig 4C). The cecum infected with 1 × 10^6^ *E. histolytica* trophozoites showed increased levels of MPO [27], while 1 × 10^5^ *E. histolytica* and *E. dispar*–challenged ceca did not (Fig 4C). Consistent with the MPO and CXCL1 results, we further examined neutrophil infiltration by anti-Ly6G immunostaining. Paraffin sections of cecal tissues collected at day 7 post-infection from sham-, 1 × 10⁶ *E. histolytica*–, and 1 × 10⁶ *E. dispar*–infected mice were analyzed (S1 Fig). As expected, infiltrating neutrophils were detected in *E. histolytica*–infected mice, whereas no positive signal was observed in sham- or *E. dispar*–infected samples.

## Discussion

This study provides the first insights into intestinal immune responses to *E. dispar*. Although the existence of nonpathogenic *Entamoeba* species had been debated since the early 20th century [Brumpt, 1925, [26]], *E. dispar* was formally recognized by Clark et al. in 1993 [26]. Since then, it has served as a comparative model for dissecting the pathogenic mechanisms of *E. histolytica*.

Using the newly isolated *E. dispar* NA442 strain, we characterized host responses in a murine cecal infection model. Despite the inability to axenize the strain, comparison with sham and *P. putida* controls showed that NA442 induced only mild inflammation while persisting for at least one week (Figs 2 and 3). In addition, natural human infections occur in the presence of complex intestinal microbiota, which may further influence parasite physiology and host immune responses. Therefore, the present monoxenic model may not fully recapitulate host–microbiota–parasite interactions occurring during human colonization. In contrast, *E. histolytica* infection resulted in strong neutrophil recruitment, consistent with prior studies, likely driven by NLRP3 inflammasome activation through Gal/GalNAc-lectin adhesion and the cysteine protease EhCP-A5 (Figs 3 and 4) [9,28–30]. This pathway promotes IL-1β release, NF-κB activation, and subsequent CXCL1 production.

Distinct from *E. histolytica*, NA442 infection caused epithelial thickening and a modest increase in fecal lipocalin-2 without detectable CXCL1 expression (Figs 3 and 4). Because lipocalin-2 is commonly used as a surrogate marker of neutrophil activity, the absence of CXCL1 suggests activation of an alternative regulatory program rather than typical neutrophil recruitment. Supporting this interpretation, MPO levels were elevated only in *E. histolytica*–infected tissues, indicating minimal neutrophil accumulation in NA442-infected mice. IL-10, a key anti-inflammatory cytokine, was undetectable in both infection settings (detection limit of the kit: 3.0 pg/mL; S2 Fig), further implying that *E. dispar* engages unique signaling pathways that limit mucosal inflammation despite transient colonization. Consistent with these observations, although a transient delay in body weight recovery was observed in mice inoculated with 1 × 10^5^ *E. dispar*, this effect was not reproduced in the 1 × 10⁶ group and was not associated with corresponding inflammatory responses. Therefore, the biological significance of this finding remains uncertain.

These findings are consistent with previous studies suggesting that intestinal responses to *Entamoeba* infection are regulated not only by neutrophil recruitment, but also by epithelial-derived homeostatic pathways. In our model, the modest induction of CXCL1 and MPO despite detectable epithelial changes and lipocalin-2 secretion may therefore reflect a regulated mucosal response rather than strong inflammatory activation. Additional studies focusing on epithelial signaling, macrophage responses, and adaptive immunity will help clarify the mechanisms underlying asymptomatic *E. dispar* colonization.

*E. dispar* likely adheres via Gal/GalNAc-lectin but lacks expression of EdCP-A5, which may prevent NF-κB activation and IL-1β–dependent CXCL1 induction [31]. Because lipocalin-2 is associated with tissue remodeling and antibacterial defense, the epithelial thickening and modest lipocalin-2 increase observed during NA442 infection likely reflect mild epithelial disturbance and enhanced exposure to luminal bacteria [32,33]. These findings support a predominantly anti-inflammatory environment that limits excessive mucosal immune activation.

The C57BL/6 background used in this study is widely regarded as an acute *E. histolytica* infection model, as parasites are typically cleared within one week [9]. Here, we employed the C57BL/6NCrSlc substrain after evaluating several mouse strains to establish a reproducible intestinal colonization model suitable for comparative analysis of host responses to *E. histolytica* and *E. dispar*. C57BL/6N mice are reported to exhibit attenuated inflammatory responses compared with C57BL/6J mice, including reduced TNF-α, IFN-γ, and viperin expression and increased IL-10 production [34–38]. Although prolonged *E. histolytica* persistence was observed in C57BL/6NCrSlc mice, canonical pathogenic features of amoebic colitis, including epithelial thickening, goblet cell expansion, lipocalin-2 and CXCL1 induction, and neutrophil recruitment, were still robustly reproduced. We nevertheless acknowledge that more immunocompetent or genetically diverse hosts may exhibit stronger neutrophil recruitment, chemokine induction, or more rapid parasite clearance.

In contrast, earlier work by Shimokawa et al. demonstrated that the axenic *E. dispar* strain AS16IR [20] was eliminated from multiple mouse strains, including both resistant C57BL/6 and susceptible CBA/J backgrounds [12]. This observation suggests that host inflammatory permissiveness alone is insufficient to explain colonization outcomes. Furthermore, the discrepancy between AS16IR and NA442 likely reflects differences in nutritional and metabolic adaptation. Whereas AS16IR proliferates axenically, NA442 requires co-culture with commensal bacteria, suggesting a dependence on host-associated microbial metabolites. Such metabolic divergence may explain strain-specific variation in colonization capacity and host interaction, and underscores the value of NA442 as a biologically relevant *E. dispar* model.

This study reports the first successful isolation of *E. dispar* NA442 from an asymptomatic HIV-positive individual and its establishment in monoxenic culture in Japan. Although *E. histolytica* infections occur domestically, *E. dispar* has previously been reported only in imported cases, highlighting the rarity of this finding. NA442 was detected incidentally during evaluation for *Pneumocystis* *pneumonia*, and the patient exhibited no gastrointestinal symptoms. Genotyping showed that NA442 shares an identical tRNA STR D-A pattern with a stool isolate from Thailand (Ed10DA) [25], which is notable given the high STR diversity typically observed in *E. dispar*. Indeed, prior surveys from Mexico and Thailand reported 15 and 13 D-A patterns among relatively small sample sets, underscoring the broad genetic heterogeneity of this species [25,39]. The match between strains from Japan and Thailand may reflect regional epidemiology, as both *E. histolytica* and *E. dispar* are endemic in parts of Thailand, where environmental conditions and high HIV prevalence may facilitate transmission [40,41]. Nevertheless, the route of infection in this case remains uncertain, and factors such as limited past sexual activity, contact with domestic animals, and his immunocompromised status may have contributed to colonization or persistence.

Although *E. dispar* is typically considered nonpathogenic, symptomatic outbreaks, such as the case in New York, suggest that strain-dependent virulence variation exists [42]. The identification of NA442 therefore highlights the need for continued molecular surveillance and for isolating additional *E. dispar* strains to better define the ecological, epidemiological, and immunological diversity within the *Entamoeba* genus.

### Limitations of this study

Despite the strengths of this study, several limitations should be acknowledged. First, because NA442 currently requires monoxenic cultivation with *P. putida*, the present observations may partly reflect physiological states associated with bacterial co-culture, even though appropriate bacterial controls were included. Second, we used the C57BL/6NCrSlc mouse background, which exhibits attenuated inflammatory signaling compared with the widely used C57BL/6J strain; thus, inflammatory responses to *E. dispar* may be underestimated in this system. Third, although NA442 persisted in the murine cecum for up to three weeks, the natural duration of *E. dispar* colonization in humans is believed to be substantially longer, often occurring as asymptomatic long-term carriage. Compared with this epidemiological evidence, our observation window in mice may represent only the early phase of colonization, and longer-term host-parasite dynamics, including potential adaptation of the parasite or mucosal remodeling, may not have been fully captured. Finally, our conclusions are based on a single clinical isolate (NA442); therefore, the observed immune responses may be strain-specific and may not fully represent the diversity of *E. dispar*. Additional *E. dispar* strains will be essential to determine whether the immune profile observed here is generalizable across species-wide genetic and phenotypic diversity. Nonetheless, NA442 represents a biologically relevant *E. dispar* model and provides a foundation for future comparative studies of virulence and host immune modulation in *Entamoeba* species.

### Conclusions

Finally, this study not only established a valuable *E. dispar* strain for comparative pathogenesis research but also highlights a possible epidemiological change in *Entamoeba* infections in Japan. Continued surveillance will be important to determine whether *E. dispar* infection may emerge more broadly within the Japanese population.

## Supporting information

S1 Fig The representative anti-Ly6G antibody-stained section on day 7. Paraffin sections of Sham-, 1 × 10^6^ *E. histolytica*–, or 1 × 10^6^ *E. dispar*–infected cecum were stained with an anti-Ly6G antibody, followed by HRP-DAB detection and hematoxylin counterstaining. Arrow heads indicate anti-Ly6G positive neutrophils. Scale bar, 500 µm.
(TIF)

S2 Fig Evaluation of IL-10 secretion in cecum. IL-10 levels were measured in cecal tissue lysates prepared from 24-hours after infection by ELISA and normalized to total protein concentration.
(TIF)
