# Supplementary material for: Evaluation of intestinal immune responses to Entamoeba dispar using the first domestic clinical isolate in Japan
Source: PLoS Negl Trop Dis. 2026 Aug 6;20(8):e0014507. doi: 10.1371/journal.pntd.0014507 (PMC13446667; doi:10.1371/journal.pntd.0014507)
Supplement: Supplementary file 1 [file pntd.0014507.s004.docx]

Response to reviewers, PNTD-D-25-02263

We thank the reviewers for their insightful and constructive comments.

We have carefully revised the manuscript in accordance with the suggestions and provide point-by-point responses below. Adding to the comments from reviewers, we added the 'Limitations of the Study' section in the Discussion following an editor's suggestion.

We have attached both the cleaned and tracked-changes versions of the manuscripts. The page numbers indicated in this document refer to the cleaned text.

***Summary and General Comments*** *Use this section to provide overall comments, discuss strengths/weaknesses of the study, novelty, significance, general execution and scholarship. You may also include additional comments for the author, including concerns about dual publication, research ethics, or publication ethics. If requesting major revision, please articulate the new experiments that are needed.*

*Reviewer #1: Important "tour de force" where one of the first infections in a human with E. disbar is characterized in the patient and in a mouse model.*

*Reviewer #2: Summary:
This study reports the first domestically acquired Entamoeba dispar infection in Japan and establishes a novel monoxenic clinical isolate (NA442). The authors demonstrate that E. dispar can transiently colonize the intestine while eliciting only minimal epithelial stress and negligible neutrophil-driven inflammation, in stark contrast to E. histolytica. The work provides a valuable experimental platform to dissect host–parasite interactions underlying non-pathogenic intestinal colonization.

Significance:
1. Demonstrating limited mucosal inflammation despite transient intestinal colonization provides important insight into mechanisms distinguishing pathogenic and non-pathogenic Entamoeba species.
2. The epidemiological finding that E. dispar may be circulating undetected in Japan is significant and holds clear public health relevance.

Major Comments:
1. The conclusions regarding E. dispar-specific immune responses are based on a single clinical isolate (NA442). Given the documented genetic and phenotypic heterogeneity within the species, the authors should explicitly frame their conclusions as potentially strain-specific rather than representative of the species as a whole. While the inclusion of additional isolates is not required for this publication, this limitation should be more clearly articulated.*

**Response:** We thank the reviewer for this important comment. We have now explicitly stated that our conclusions are based on a single clinical isolate (NA442) and may therefore reflect strain-specific characteristics rather than species-wide properties. We have modified the sentence in the Discussion section, as shown below (lines 486-488), “Finally, our conclusions are based on a single clinical isolate (NA442); therefore, the observed immune responses may be strain-specific and may not fully represent the diversity of *E. dispar*.”

*2. Line 353: The NA442 isolate requires co-culture with Pseudomonas putida, raising the possibility that bacterial metabolites contribute to epithelial stress signals (e.g., lipocalin-2 induction). Although appropriate bacterial controls were utilized, the authors should discuss how this monoxenic dependency might limit the extrapolation of these results to natural human infections involving complex, polymicrobial microbiota.*

**Response:** We appreciate this comment. Although bacterial-only controls were included, the use of monoxenically cultured NA442 may still influence host responses. Because NA442 currently requires co-culture with *Pseudomonas putida* for in vitro maintenance, bacterial association necessarily alters the physiological state of *E. dispar*, including its metabolism, growth, surface properties, and secretory activity, and may thereby modulate epithelial stress or innate immune responses in ways that are not fully reproduced by bacterial-only controls. In addition, interactions between trophozoites and associated bacteria may generate composite stimuli that differ from those occurring in axenic conditions. Furthermore, natural human infections occur in the presence of complex intestinal microbiota, which may further influence parasite physiology and host immune responses. Therefore, the present monoxenic model may not fully recapitulate host–microbiota–parasite interactions during human colonization, and the observed responses should be interpreted as host responses to monoxenically cultured NA442 rather than to axenic *E. dispar* alone. We added these sentences in Line 390-394, “In addition, natural human infections occur in the presence of complex intestinal microbiota, which may further influence parasite physiology and host immune responses. Therefore, the present monoxenic model may not fully recapitulate host–microbiota–parasite interactions occurring during human colonization.”.

*3. Line 361: The immunological analysis focuses heavily on neutrophil-associated markers (CXCL1, MPO, lipocalin-2). Additional discussion is warranted regarding other potential immune pathways—such as epithelial-derived signaling, macrophage activation, or adaptive immune responses—that may facilitate asymptomatic colonization but were not examined in this study.*
**Response:** We appreciate this important comment. In the present study, we primarily focused on early neutrophil-associated and epithelial stress-related markers, including CXCL1, MPO, and lipocalin-2, as indicators of acute innate immune activation. However, we agree that additional immune pathways may contribute to the establishment of asymptomatic colonization by *E. dispar*.

Previous studies of experimental amebiasis have demonstrated that host responses to *Entamoeba* infection are not solely determined by neutrophil recruitment, but also involve epithelial-derived regulatory pathways, IL-10-dependent immune homeostasis, dendritic cell activation, and microbiota-associated innate conditioning. In particular, resistance or susceptibility to intestinal amebiasis has been linked to nonhematopoietic compartments, including epithelial responses that regulate mucosal integrity and inflammatory tone. In addition, microbiota-driven IL-17A signaling and bone marrow-derived innate immune conditioning have been shown to modulate intestinal responses to *Entamoeba* infection.

In the present model, the relatively modest induction of CXCL1 and MPO despite detectable epithelial changes and lipocalin-2 secretion may suggest that NA442 induces a limited or regulated mucosal response rather than strong inflammatory activation. Such responses could involve epithelial barrier adaptation, macrophage-mediated regulation, tolerogenic innate pathways, or adaptive immune mechanisms that were not examined in this study. Further investigation of cytokine networks, epithelial signaling pathways, macrophage responses, and adaptive immunity will therefore be important to better understand how *E. dispar* establishes long-term asymptomatic colonization.

We added these considerations to the Discussion section (Line 413–420).

*4. Line 376: The authors utilized C57BL/6NCrSlc mice, a sub-strain known to exhibit attenuated inflammatory responses compared to C57BL/6J. This choice may lead to an underestimation of mucosal immune activation. Please provide a stronger scientific justification for selecting this specific sub-strain and discuss how the results might differ in a more immunocompetent or diverse host. Additionally, clarify if this choice was specifically intended to observe longer-term E. histolytica colonization.*
**Response:** Thank you for this important comment. We agree that C57BL/6NCrSlc mice exhibit relatively attenuated inflammatory responses compared with C57BL/6J mice, and therefore the magnitude of mucosal immune activation observed in this study may be underestimated.

The selection of this substrain was not intended solely to artificially prolong *Entamoeba* colonization, but rather to establish a stable intestinal infection model suitable for evaluating early host responses to the newly isolated *E. dispar* strain. Importantly, previous studies demonstrated that host inflammatory responsiveness alone does not determine intestinal colonization by *Entamoeba* species (Shimokawa et al., JID, 2012). In established murine amebiasis models, C57BL/6 mice are resistant to *E. histolytica* infection, whereas CBA/J mice are susceptible and permit prolonged colonization. However, *E. dispar* failed to establish infection in either resistant or susceptible mouse backgrounds. These findings suggest that successful intestinal persistence depends not only on host immune permissiveness but also on parasite-specific biological properties.

Therefore, although the moderate inflammatory background of C57BL/6NCrSlc mice may have facilitated observation of early colonization dynamics, the persistence of the NA442 strain cannot be explained solely by reduced host inflammatory responses. We nevertheless acknowledge that more immunocompetent or genetically diverse hosts may exhibit stronger neutrophil recruitment, chemokine induction, or more rapid parasite clearance. We have added this point to the Discussion section (lines 428-440).

Minor Comments:
*1. Please link statistical methods more explicitly to specific figures within the Methods section or figure legends.*
**Response:** Thank you for the comment. In the revised manuscript, we have clarified the statistical methods in the corresponding figure legends to more explicitly link each analysis to the relevant figures.

*2. Terminology describing “inflammation” versus “epithelial stress” should be used consistently to avoid over-interpretation of lipocalin-2 data.*

**Response:** Thank you for your comment. We agree that lipocalin-2 alone is insufficient to define inflammatory responses and that interpretation of this marker without additional inflammatory evidence may overestimate the degree of inflammation. Therefore, we revised the manuscript to consistently use the term “epithelial stress response” when referring to lipocalin-2–associated changes and to avoid describing these responses as “inflammation” unless supported by additional evidence such as neutrophil-associated responses or tissue damage. Accordingly, we modified the relevant sentences throughout the manuscript, including the following: in the Author Summary, “*E. dispar* triggered epithelial stress response, with limited neutrophil-associated responses and no evidence of tissue damage ” (lines 90–92), and in the Results section, “These results demonstrate that same count of *E. histolytica* and *E. dispar* induced epithelial stress response, and infection of *E. histolytica* induces higher level of lipocalin than that of *E. dispar* ” (lines 352–355).

*3. Regarding Figure 3C, please detail the anatomical consistency of the cecal sections used for measurement to ensure the reliability of the quantitative thickness data.*

**Response:** Thank you for the comment. We added a description of the measurement method in the Materials and Methods section (lines 230-243), including the use of anatomically comparable cecal regions and multiple measurement sites per mouse to minimize variability due to sectioning position.

*4. Line 365: Please provide the detection limits for the IL-10 ELISA or include the raw data (even if negative) in the Supplementary Materials to support this claim.*

**Response:** Thank you for the comment. We added the IL-10 data as Supplementary S2 Fig. and included the detection limit of the ELISA kit (3.0 pg/mL) in the Discussion section (line 406).

*Reviewer #3: The authors present the first detailed immunological analysis of cecal infection in mice over a 3-week period using the genotypically verified E. dispar strain NA442. The study specifically examines neutrophils and their associated effectors, including CXCL1 and MPO, which show no infection-dependent changes, recruitment alterations, or signs of inflammation, in contrast to infections with the human pathogen E. histolytica. Interestingly, only the epithelial stress marker lipocalin-2 indicated a potential involvement of the gut endothelium. This work provides the first comprehensive insight into the host immune response to the apathogenic E. dispar, highlighting key differences from pathogenic amebic infections and offering a foundation for further mechanistic studies of host–pathogen interactions in the gut.
Major Comments:
While the study addresses an important and understudied question and presents a promising experimental approach, several issues should be addressed to strengthen the manuscript’s scientific rigor and interpretability.
1. The detailed description of the isolate’s origin and genotypic identification is thorough, but this section reads almost like a case report and is somewhat lengthy. Condensing it would help maintain focus on the immunological findings and strengthen the overall narrative.*

**Response:** Thank you for the comment. Initially, we aimed to document the first domestically acquired case of E. dispar in Japan and therefore included detailed information regarding the isolate. However, we agree with the reviewer that this section was overly detailed and could distract from the main immunological findings. Accordingly, we condensed the “Clinical case presentation” section in the Results (lines 264-282) by simplifying the clinical and strain-related descriptions, reducing the word count from lines 243 to 213.

*2. A major concern is the discrepancy in inoculum doses between E. histolytica and E. dispar. The higher dose used for E. histolytica substantially limits the scientific comparability between the two infections. I regret that equivalent doses were not used, as repeating the experiments with matched inocula (e.g., 1×10⁵) would provide more robust and interpretable comparisons. Corresponding immunostainings and analyses should be repeated using these matched samples.*

**Response:** Thank you for the comment. We agree that the difference in inoculum size limited the direct comparability between the *E. histolytica* and *E. dispar* infection models. To address this concern, we repeated the key analyses using mice infected with 1×10^6^ *E. dispar* and 1×10^5^ *E. histolytica* trophozoites and incorporated these data into the revised manuscript. Specifically, we added the data for body weight dynamics (Fig. 2A), gross cecal appearance (Fig. 2B), infection rate on day7 (Table 1), lipocalin-2 secretion (Fig. 3A), histological analysis (Fig. 3B), mucosal thickness measurements (Fig. 3C), goblet cell quantification (Fig. 4A), CXCL1 levels (Fig. 4B), and MPO levels (Fig. 4C).

*3. The E. histolytica infection model appears robust and particularly interesting, as intestinal ligation does not seem necessary to establish infection. This model offers a valuable opportunity to investigate the murine immune response specifically to E. histolytica intestinal infection and could serve as a reference for future mechanistic studies.*

**Response:** Thank you for this positive comment. We agree that the present ligation-free murine cecal infection model provides a useful experimental platform for investigating host immune responses to intestinal *E. histolytica* infection. We believe this model may facilitate future mechanistic studies of host–parasite interactions and mucosal immune regulation during amebic infection.

*Minor Comments:
1. Line 93: Please expand on the connection between leptin receptor mutation and neutrophil function, providing mechanistic or literature-based context.*

**Response:** Thank you for your comment. The precise molecular mechanism linking the leptin receptor Q223R mutation to impaired neutrophil responses has not been fully clarified. However, previous studies by Duggal et al. and Naylor et al. demonstrated that: (i) the leptin receptor Q223R variant, originally identified through GWAS analysis, is associated with increased susceptibility to intestinal amebiasis; (ii) neutrophil infiltration into the intestinal mucosa is reduced in mutant mice; (iii) chemokine production in the cecum is comparable between wild-type and mutant mice; and (iv) neutrophils from mutant mice exhibit reduced chemotactic responses to leptin. These findings suggest that impaired leptin-dependent neutrophil chemotaxis, rather than reduced chemokine induction, contributes to defective mucosal neutrophil recruitment.

To clarify this point, we revised the sentence in the Introduction as follows: “Host genetic studies have linked leptin receptor Q233R mutation to susceptibility to intestinal amebiasis, highlighting the importance of neutrophil recruitment in restricting E. histolytica colonization in the intestine, potentially due to impaired leptin-dependent neutrophil chemotaxis (5,6).” (lines 107–110).

*2. Line 96: Provide a more detailed description of the mouse model used here, including a comparison with prior E. histolytica infection models (e.g., cecum ligation followed by injection), which exhibited robust infection. Highlighting these differences will clarify the model’s relevance and novelty.*

**Response:** Thank you for this helpful comment. We agree that additional clarification regarding the experimental model and its relationship to previous intestinal amebiasis models would improve the manuscript.

Since the establishment of the murine intracecal inoculation model in 2002, non-ligated cecal infection models have been widely used to evaluate intestinal colonization by *E. histolytica*. Importantly, these studies demonstrated strong mouse strain dependency, in which C57BL/6 (C57BL/6J) mice were relatively resistant and rapidly cleared infection, whereas C3H/HeJ and CBA mice were more susceptible and supported prolonged colonization for up to several months. However, to our knowledge, stable intestinal colonization by *E. dispar* has not been successfully demonstrated in these conventional models.

The present study differs from previous models in that both *E. histolytica* and the newly isolated *E. dispar* strain NA442 were able to transiently colonize the intestine under the same experimental conditions in C57BL/6NCrSlc mice, enabling comparative evaluation of early mucosal responses between pathogenic and non-pathogenic *Entamoeba* species. We believe this represents an important feature and utility of the present model.

To clarify these points, we modified the relevant descriptions in the Introduction (lines 110–118).

*3. Line 240: Include normal CD4⁺ T cell counts as a reference to facilitate interpretation of deviations observed in the infected groups.*
**Response:** Thank you for pointing out the lack of this important information. We revised the sentence in the Results section (Lines 266–268) as follows: “He had previously maintained viral suppression on cART, with a CD4 count of 150 cells/µL at his last routine visit (normal reference range: 500-1500 cells/µL; below the critical threshold of 200 cells/µL for opportunistic infections).”

*Figures:
• Figures 2 & 3: The use of a higher inoculum for E. histolytica compared to E. dispar + P. perfringens strongly affects comparability. Again, I regret that identical doses were not used, as this would allow for more meaningful conclusions.*

**Response:** Thank you for your comment. As addressed in Major Comment #2, we repeated the experiments using 1 × 10^6^ *E. dispar,* co-cultivating with *P. putida* and 1 × 10^5^ *E. histolytica* trophozoites in the revised manuscript. (We note that the bacterium used in these experiments was *P. putida*.) These additional experiments consistently demonstrated that *E. dispar* induced a substantially weaker inflammatory response compared with *E. histolytica*.

*• Immunostaining for neutrophils (anti-Ly6G) is recommended for both E. dispar and E. histolytica infections, as the samples appear to be available. This would strengthen the analysis of innate immune responses.*

**Response:** Thank you for your comment. We added the immunostaining data to S1 Fig. As expected, anti-Ly6G antibody staining specifically detected infiltrated neutrophils in the cecal tissues infected with 1 × 10^6^ *E. histolytica* trophozoites.

*• Figure 4: If serum from these mice is available, analysis of a broader cytokine panel would provide a more comprehensive picture of the immune response to E. dispar, enhancing the manuscript’s mechanistic insight.*

**Response:** Thank you very much for this valuable suggestion. Unfortunately, serum samples were not preserved from this series of experiments. However, we agree that a broader cytokine analysis would provide important additional insight into the host immune response to *E. dispar*. At present, our data are insufficient to fully define the systemic immune response induced by *E. dispar*. To gain deeper insight into *E. dispar*–host interactions, we plan to perform comprehensive cytokine profiling in future studies.
