# Supplementary material for: Evaluation of intestinal immune responses to Entamoeba dispar using the first domestic clinical isolate in Japan
Source: PLoS Negl Trop Dis. 2026 Aug 6;20(8):e0014507. doi: 10.1371/journal.pntd.0014507 (PMC13446667; doi:10.1371/journal.pntd.0014507)
Supplement: Peer review history

# Peer review: Decision Letter 0

**DOI:** 10.1371/journal.pntd.0014507.r001

**Authors:** Sarman Singh

7 Jan 2026

-->PNTD-D-25-02263

Evaluation of intestinal immune responses to Entamoeba dispar using the first domestic clinical isolate in Japan

PLOS Neglected Tropical Diseases

Dear Dr. Nakada-Tsukui,

Thank you for submitting your manuscript to PLOS Neglected Tropical Diseases. After careful consideration, we feel that it has merit but does not fully meet PLOS Neglected Tropical Diseases's publication criteria as it currently stands. Therefore, we invite you to submit a revised version of the manuscript that addresses the points raised during the review process.

Please submit your revised manuscript within by Mar 08 2026 11:59PM. If you will need more time than this to complete your revisions, please reply to this message or contact the journal office at plosntds@plos.org. When you're ready to submit your revision, log on to https://www.editorialmanager.com/pntd/ and select the 'Submissions Needing Revision' folder to locate your manuscript file.

Please include the following items when submitting your revised manuscript:

* A letter that responds to each point raised by the editor and reviewer(s). You should upload this letter as a separate file labeled 'Response to Reviewers'. This file does not need to include responses to any formatting updates and technical items listed in the 'Journal Requirements' section below.

* A marked-up copy of your manuscript that highlights changes made to the original version. You should upload this as a separate file labeled 'Revised Manuscript with Track Changes'.

* An unmarked version of your revised paper without tracked changes. You should upload this as a separate file labeled 'Manuscript'.

If you would like to make changes to your financial disclosure, competing interests statement, or data availability statement, please make these updates within the submission form at the time of resubmission. Guidelines for resubmitting your figure files are available below the reviewer comments at the end of this letter.

We look forward to receiving your revised manuscript.

Kind regards,

Sarman Singh, MD, FRSC, FRCP

Section Editor

PLOS Neglected Tropical Diseases

Sarman Singh

Section Editor

PLOS Neglected Tropical Diseases

Shaden Kamhawi

co-Editor-in-Chief

PLOS Neglected Tropical Diseases

orcid.org/0000-0003-4304-636XX

Paul Brindley

co-Editor-in-Chief

PLOS Neglected Tropical Diseases

orcid.org/0000-0003-1765-0002

**Additional Editor Comments:**

Even though one reviewer has been very liberal but other reviewers, especially Reviewer 3 has raised pertient question regardng the conclusions based on a single clinical isolate (NA442), and I tend to agree with the reviewer. I would strongly advise the authors to include a dedicated paragraph on the limitations of the study.

**Journal Requirements:**

1) Please ensure that the CRediT author contributions listed for every co-author are completed accurately and in full.

At this stage, the following Authors/Authors require contributions: Yumiko Saito-Nakano, Akira Kawashima, Seiki Kobayashi, Koji Watanabe, Tetsuro Kawano-Sugaya, Shinji Izumiyama, and Kumiko Nakada-Tsukui. Please ensure that the full contributions of each author are acknowledged in the "Add/Edit/Remove Authors" section of our submission form.

The list of CRediT author contributions may be found here: https://journals.plos.org/plosntds/s/authorship#loc-author-contributions

2) We ask that a manuscript source file is provided at Revision. Please upload your manuscript file as a .doc, .docx, .rtf or .tex. If you are providing a .tex file, please upload it under the item type u2018LaTeX Source Fileu2019 and leave your .pdf version as the item type u2018Manuscriptu2019.

3) We noticed that you used the phrase 'data not shown' in the manuscript. We do not allow these references, as the PLOS data access policy requires that all data be either published with the manuscript or made available in a publicly accessible database. Please amend the supplementary material to include the referenced data or remove the references.

4) We do not publish any copyright or trademark symbols that usually accompany proprietary names, eg ©,  ®, or TM  (e.g. next to drug or reagent names). Therefore please remove all instances of trademark/copyright symbols throughout the text, including:

- TM on page: 15.

5) Please upload all main figures as separate Figure files in .tif or .eps format. For more information about how to convert and format your figure files please see our guidelines:

https://journals.plos.org/plosntds/s/figures

6) Some material included in your submission may be copyrighted. According to PLOSu2019s copyright policy, authors who use figures or other material (e.g., graphics, clipart, maps) from another author or copyright holder must demonstrate or obtain permission to publish this material under the Creative Commons Attribution 4.0 International (CC BY 4.0) License used by PLOS journals. Please closely review the details of PLOSu2019s copyright requirements here: PLOS Licenses and Copyright. If you need to request permissions from a copyright holder, you may use PLOS's Copyright Content Permission form.

Please respond directly to this email and provide any known details concerning your material's license terms and permissions required for reuse, even if you have not yet obtained copyright permissions or are unsure of your material's copyright compatibility. Once you have responded and addressed all other outstanding technical requirements, you may resubmit your manuscript within Editorial Manager.

Potential Copyright Issues:

- Please confirm (a) that you are the photographer of Figure 2B., or (b) provide written permission from the photographer to publish the photo(s) under our CC BY 4.0 license.

**Reviewers' Comments:**

Reviewer's Responses to Questions

**Key Review Criteria Required for Acceptance?**

As you describe the new analyses required for acceptance, please consider the following:

**Methods**

-Are the objectives of the study clearly articulated with a clear testable hypothesis stated?

-Is the study design appropriate to address the stated objectives?

-Is the population clearly described and appropriate for the hypothesis being tested?

-Is the sample size sufficient to ensure adequate power to address the hypothesis being tested?

-Were correct statistical analysis used to support conclusions?

-Are there concerns about ethical or regulatory requirements being met?

Reviewer #1: Methods are clearly described and appropriate for testing the hypothesis that E. disparity infection is minimally invasive.

Reviewer #2: Accept

Reviewer #3: good, no comments

**********

**Results**

-Does the analysis presented match the analysis plan?

-Are the results clearly and completely presented?

-Are the figures (Tables, Images) of sufficient quality for clarity?

Reviewer #1: Results are clearly and completely presented

Reviewer #2: Accept

Reviewer #3: good, no comments

**********

**Conclusions**

-Are the conclusions supported by the data presented?

-Are the limitations of analysis clearly described?

-Do the authors discuss how these data can be helpful to advance our understanding of the topic under study?

-Is public health relevance addressed?

Reviewer #1: Conclusions are supported by the data

Reviewer #2: “Minor Revision” The conclusions regarding E. dispar-specific immune responses are based on a single clinical isolate (NA442). Given the documented genetic and phenotypic heterogeneity within the species, the authors should explicitly frame their conclusions as potentially strain-specific rather than representative of the species as a whole. While the inclusion of additional isolates is not required for this publication, this limitation should be more clearly articulated.

Reviewer #3: not really, as the comparability with two differnet dosgaes of a necessary control are partially not correct

**********

**Editorial and Data Presentation Modifications?**

Use this section for editorial suggestions as well as relatively minor modifications of existing data that would enhance clarity. If the only modifications needed are minor and/or editorial, you may wish to recommend “Minor Revision” or “Accept”.

Reviewer #1: none

Reviewer #2: Accept

Reviewer #3: see comments below

**********

**Summary and General Comments**

Use this section to provide overall comments, discuss strengths/weaknesses of the study, novelty, significance, general execution and scholarship. You may also include additional comments for the author, including concerns about dual publication, research ethics, or publication ethics. If requesting major revision, please articulate the new experiments that are needed.

Reviewer #1: Important "tour de force" where one of the first infections in a human with E. disbar is characterized in the patient and in a mouse model.

Reviewer #2: Summary:

This study reports the first domestically acquired Entamoeba dispar infection in Japan and establishes a novel monoxenic clinical isolate (NA442). The authors demonstrate that E. dispar can transiently colonize the intestine while eliciting only minimal epithelial stress and negligible neutrophil-driven inflammation, in stark contrast to E. histolytica. The work provides a valuable experimental platform to dissect host–parasite interactions underlying non-pathogenic intestinal colonization.

Significance:

1. Demonstrating limited mucosal inflammation despite transient intestinal colonization provides important insight into mechanisms distinguishing pathogenic and non-pathogenic Entamoeba species.

2. The epidemiological finding that E. dispar may be circulating undetected in Japan is significant and holds clear public health relevance.

Major Comments:

1. The conclusions regarding E. dispar-specific immune responses are based on a single clinical isolate (NA442). Given the documented genetic and phenotypic heterogeneity within the species, the authors should explicitly frame their conclusions as potentially strain-specific rather than representative of the species as a whole. While the inclusion of additional isolates is not required for this publication, this limitation should be more clearly articulated.

2. Line 353: The NA442 isolate requires co-culture with Pseudomonas putida, raising the possibility that bacterial metabolites contribute to epithelial stress signals (e.g., lipocalin-2 induction). Although appropriate bacterial controls were utilized, the authors should discuss how this monoxenic dependency might limit the extrapolation of these results to natural human infections involving complex, polymicrobial microbiota.

3. Line 361: The immunological analysis focuses heavily on neutrophil-associated markers (CXCL1, MPO, lipocalin-2). Additional discussion is warranted regarding other potential immune pathways—such as epithelial-derived signaling, macrophage activation, or adaptive immune responses—that may facilitate asymptomatic colonization but were not examined in this study.

4. Line 376: The authors utilized C57BL/6NCrSlc mice, a sub-strain known to exhibit attenuated inflammatory responses compared to C57BL/6J. This choice may lead to an underestimation of mucosal immune activation. Please provide a stronger scientific justification for selecting this specific sub-strain and discuss how the results might differ in a more immunocompetent or diverse host. Additionally, clarify if this choice was specifically intended to observe longer-term E. histolytica colonization.

Minor Comments:

1. Please link statistical methods more explicitly to specific figures within the Methods section or figure legends.

2. Terminology describing “inflammation” versus “epithelial stress” should be used consistently to avoid over-interpretation of lipocalin-2 data.

3. Regarding Figure 3C, please detail the anatomical consistency of the cecal sections used for measurement to ensure the reliability of the quantitative thickness data.

4. Line 365: Please provide the detection limits for the IL-10 ELISA or include the raw data (even if negative) in the Supplementary Materials to support this claim.

Reviewer #3: The authors present the first detailed immunological analysis of cecal infection in mice over a 3-week period using the genotypically verified E. dispar strain NA442. The study specifically examines neutrophils and their associated effectors, including CXCL1 and MPO, which show no infection-dependent changes, recruitment alterations, or signs of inflammation, in contrast to infections with the human pathogen E. histolytica. Interestingly, only the epithelial stress marker lipocalin-2 indicated a potential involvement of the gut endothelium. This work provides the first comprehensive insight into the host immune response to the apathogenic E. dispar, highlighting key differences from pathogenic amebic infections and offering a foundation for further mechanistic studies of host–pathogen interactions in the gut.

Major Comments:

While the study addresses an important and understudied question and presents a promising experimental approach, several issues should be addressed to strengthen the manuscript’s scientific rigor and interpretability.

1. The detailed description of the isolate’s origin and genotypic identification is thorough, but this section reads almost like a case report and is somewhat lengthy. Condensing it would help maintain focus on the immunological findings and strengthen the overall narrative.

2. A major concern is the discrepancy in inoculum doses between E. histolytica and E. dispar. The higher dose used for E. histolytica substantially limits the scientific comparability between the two infections. I regret that equivalent doses were not used, as repeating the experiments with matched inocula (e.g., 1×10⁵) would provide more robust and interpretable comparisons. Corresponding immunostainings and analyses should be repeated using these matched samples.

3. The E. histolytica infection model appears robust and particularly interesting, as intestinal ligation does not seem necessary to establish infection. This model offers a valuable opportunity to investigate the murine immune response specifically to E. histolytica intestinal infection and could serve as a reference for future mechanistic studies.

Minor Comments:

1. Line 93: Please expand on the connection between leptin receptor mutation and neutrophil function, providing mechanistic or literature-based context.

2. Line 96: Provide a more detailed description of the mouse model used here, including a comparison with prior E. histolytica infection models (e.g., cecum ligation followed by injection), which exhibited robust infection. Highlighting these differences will clarify the model’s relevance and novelty.

3. Line 240: Include normal CD4⁺ T cell counts as a reference to facilitate interpretation of deviations observed in the infected groups.

Figures:

• Figures 2 & 3: The use of a higher inoculum for E. histolytica compared to E. dispar + P. perfringens strongly affects comparability. Again, I regret that identical doses were not used, as this would allow for more meaningful conclusions.

• Immunostaining for neutrophils (anti-Ly6G) is recommended for both E. dispar and E. histolytica infections, as the samples appear to be available. This would strengthen the analysis of innate immune responses.

• Figure 4: If serum from these mice is available, analysis of a broader cytokine panel would provide a more comprehensive picture of the immune response to E. dispar, enhancing the manuscript’s mechanistic insight.

**********

PLOS authors have the option to publish the peer review history of their article (what does this mean?). If published, this will include your full peer review and any attached files.

If you choose “no”, your identity will remain anonymous but your review may still be made public.

**Do you want your identity to be public for this peer review?**  For information about this choice, including consent withdrawal, please see our Privacy Policy.

Reviewer #1: **Yes:** William A. Petri, jr

Reviewer #2: No

Reviewer #3: No

[NOTE: If reviewer comments were submitted as an attachment file, they will be attached to this email and accessible via the submission site. Please log into your account, locate the manuscript record, and check for the action link "View Attachments". If this link does not appear, there are no attachment files.]

**Figure resubmission:**

While revising your submission, we strongly recommend that you use PLOS’s NAAS tool (https://ngplosjournals.pagemajik.ai/artanalysis) to test your figure files. NAAS can convert your figure files to the TIFF file type and meet basic requirements (such as print size, resolution), or provide you with a report on issues that do not meet our requirements and that NAAS cannot fix.

-->

After uploading your figures to PLOS’s NAAS tool - https://ngplosjournals.pagemajik.ai/artanalysis, NAAS will process the files provided and display the results in the "Uploaded Files" section of the page as the processing is complete. If the uploaded figures meet our requirements (or NAAS is able to fix the files to meet our requirements), the figure will be marked as "fixed" above. If NAAS is unable to fix the files, a red "failed" label will appear above. When NAAS has confirmed that the figure files meet our requirements, please download the file via the download option, and include these NAAS processed figure files when submitting your revised manuscript.-->

**Reproducibility:**

To enhance the reproducibility of your results, we recommend that authors of applicable studies deposit laboratory protocols in protocols.io, where a protocol can be assigned its own identifier (DOI) such that it can be cited independently in the future. Additionally, PLOS ONE offers an option to publish peer-reviewed clinical study protocols. Read more information on sharing protocols at https://plos.org/protocols?utm_medium=editorial-email&utm_source=authorletters&utm_campaign=protocols

-->

# Author comment: Author response to Decision Letter 1

**DOI:** 10.1371/journal.pntd.0014507.r002

16 Jun 2026

Attachment Submitted filename: Saito-Nakano_PTPR_knt606.docx

# Peer review: Decision Letter 1

**DOI:** 10.1371/journal.pntd.0014507.r003

**Authors:** Sarman Singh

26 Jun 2026

Dear Dr Nakada-Tsukui,

We are pleased to inform you that your manuscript 'Evaluation of intestinal immune responses to Entamoeba dispar using the first domestic clinical isolate in Japan' has been provisionally accepted for publication in PLOS Neglected Tropical Diseases.

Before your manuscript can be formally accepted you will need to complete some formatting changes, which you will receive in a follow up email. A member of our team will be in touch with a set of requests.

Please note that your manuscript will not be scheduled for publication until you have made the required changes, so a swift response is appreciated.

IMPORTANT: The editorial review process is now complete. PLOS will only permit corrections to spelling, formatting or significant scientific errors from this point onwards. Requests for major changes, or any which affect the scientific understanding of your work, will cause delays to the publication date of your manuscript.

Should you, your institution's press office or the journal office choose to press release your paper, you will automatically be opted out of early publication. We ask that you notify us now if you or your institution is planning to press release the article. All press must be co-ordinated with PLOS.

Thank you again for supporting Open Access publishing; we are looking forward to publishing your work in PLOS Neglected Tropical Diseases.

Best regards,

Sarman Singh, MD, FRSC, FRCP

Section Editor

PLOS Neglected Tropical Diseases

Sarman Singh

Section Editor

PLOS Neglected Tropical Diseases

Shaden Kamhawi

co-Editor-in-Chief

PLOS Neglected Tropical Diseases

orcid.org/0000-0003-4304-636XX

Paul Brindley

co-Editor-in-Chief

PLOS Neglected Tropical Diseases

orcid.org/0000-0003-1765-0002

***********************************************************

p.p1 {margin: 0.0px 0.0px 0.0px 0.0px; line-height: 16.0px; font: 14.0px Arial; color: #323333; -webkit-text-stroke: #323333}span.s1 {font-kerning: none

Reviewer's Responses to Questions

**Key Review Criteria Required for Acceptance?**

As you describe the new analyses required for acceptance, please consider the following:

**Methods**

-Are the objectives of the study clearly articulated with a clear testable hypothesis stated?

-Is the study design appropriate to address the stated objectives?

-Is the population clearly described and appropriate for the hypothesis being tested?

-Is the sample size sufficient to ensure adequate power to address the hypothesis being tested?

-Were correct statistical analysis used to support conclusions?

-Are there concerns about ethical or regulatory requirements being met?

Reviewer #1: N/a - revised manuscript

**********

**Results**

-Does the analysis presented match the analysis plan?

-Are the results clearly and completely presented?

-Are the figures (Tables, Images) of sufficient quality for clarity?

Reviewer #1: N/a - revised manuscript

**********

**Conclusions**

-Are the conclusions supported by the data presented?

-Are the limitations of analysis clearly described?

-Do the authors discuss how these data can be helpful to advance our understanding of the topic under study?

-Is public health relevance addressed?

Reviewer #1: N/a - revised manuscript

**********

**Editorial and Data Presentation Modifications?**

Use this section for editorial suggestions as well as relatively minor modifications of existing data that would enhance clarity. If the only modifications needed are minor and/or editorial, you may wish to recommend “Minor Revision” or “Accept”.

Reviewer #1: N/a - revised manuscript

**********

**Summary and General Comments**

Use this section to provide overall comments, discuss strengths/weaknesses of the study, novelty, significance, general execution and scholarship. You may also include additional comments for the author, including concerns about dual publication, research ethics, or publication ethics. If requesting major revision, please articulate the new experiments that are needed.

Reviewer #1: The authors have comprehensively and more than adequately responded to the comments of the reviewer #3, adding to the discussion the limitations of the work (single mouse strain, single parasite isolate, bacterial co-culture) and emphasized the considerable strengths of the work including the study of the first clinical isolate of E dispar from Japan in a mouse model, with the mouse model replicating the non-invaisve and modestly inflammatory host-parasite interaction.

**********

PLOS authors have the option to publish the peer review history of their article (what does this mean?). If published, this will include your full peer review and any attached files.

If you choose “no”, your identity will remain anonymous but your review may still be made public.

**Do you want your identity to be public for this peer review?**  For information about this choice, including consent withdrawal, please see our Privacy Policy.

Reviewer #1: **Yes:** William A. Petri, Jr.

# Editor report: Acceptance letter

**DOI:** 10.1371/journal.pntd.0014507.r004

**Authors:** Sarman Singh

Dear Dr Nakada-Tsukui,

We are delighted to inform you that your manuscript, "Evaluation of intestinal immune responses to Entamoeba dispar using the first domestic clinical isolate in Japan," has been formally accepted for publication in PLOS Neglected Tropical Diseases.

We have now passed your article onto the PLOS Production Department who will complete the rest of the publication process. All authors will receive a confirmation email upon publication.

The corresponding author will soon be receiving a typeset proof for review, to ensure errors have not been introduced during production. Please review the PDF proof of your manuscript carefully, as this is the last chance to correct any scientific or type-setting errors. Please note that major changes, or those which affect the scientific understanding of the work, will likely cause delays to the publication date of your manuscript. Note: Proofs for Front Matter articles (Editorial, Viewpoint, Symposium, Review, etc...) are generated on a different schedule and may not be made available as quickly.

Soon after your final files are uploaded, the early version of your manuscript will be published online unless you opted out of this process. The date of the early version will be your article's publication date. The final article will be published to the same URL, and all versions of the paper will be accessible to readers.

For Research Articles, you will receive an invoice from PLOS for your publication fee after your manuscript has reached the completed accept phase. If you receive an email requesting payment before acceptance or for any other service, this may be a phishing scheme. Learn how to identify phishing emails and protect your accounts at https://explore.plos.org/phishing.

Thank you again for supporting open-access publishing; we are looking forward to publishing your work in PLOS Neglected Tropical Diseases.

Best regards,

Shaden Kamhawi

co-Editor-in-Chief

PLOS Neglected Tropical Diseases

Paul Brindley

co-Editor-in-Chief

PLOS Neglected Tropical Diseases
